# Milk Abscess

**Published:** 1849-11

**Authors:** 


*Milk Abscess.*—There is no accident to which the parturient female is exposed, more dreaded by herself, than what she designates a "gathered breast;" and having been once afflicted with this kind of abscess, she anticipates its return, on every occasion of her *lying in;* hence it is of importance, as well for the success and reputation of the accoucheur, as for the comfort and well being of his patient, that such an occurrence should be averted, if possible. And as the testimony of all writers upon the subject, is united in support of the opinion, that there is no phlegmonous inflammation, which takes on the suppurative process, with more certainty or rapidity, than that which attacks the female mammæ—to procure a resolution of the inflammatory action, is manifestly the first indication to be met, and the most desirable object to be gained; but experience has proved how fruitless the most active measures have been, may it not be said, in the majority of cases! Saline cathartics, local or general bleeding, as well as a variety of external applications, have been employed in the practice of the best and wisest men; but with what success, the painful history of the past abundantly testifies. The late Dr. Dewees, whose reputation in this department of science, entitles his testimony to the highest regard, recommends bathing the breast with warm vinegar, as the most successful application in his hands; to be conjoined, of course, with the usual constitutional remedies, for inviting the blood to other parts of the system, and reducing the local inflammation. It is true, that the failure to resolve the inflammation may frequently be the result of carelessness on the part of the attendant, or of injudicious management in the early stage of the malady; but the "pertinacity of its course," to use the language of Dr. Dewees, is a peculiarity of mammary inflammation, which renders it capable of resisting, in a great many cases, the most active antiphlogistic treatment. During the first few years of my practice, I had frequently to lament the ill success of remedies, which I had learned to estimate as powerful resolvents; and notwithstanding the most faithful perseverance in their application, I was frequently obliged to abandon them, and substitute supporting constitutional remedies, with such external means as were designed to aid the suppurative process. And it may not be considered presumptuous in me to say, that after repeated trials with a variety of unguents and liniments, I have abandoned them all, except the following, which I use in nearly every case of mammary abscess, and generally with entire satisfaction.

℞. Ung. Tabaci. oz. ii.
Pulv. Camph. drachm ii.
Ext. Belladon. drchm. iss. ft. ung.

The belladonna is not always used, though I do not know that it is ever inadmissable. The tobacco ointment was first introduced to my notice by Wm. J. Allinson, an apothecary of this city, who makes it in a manner somewhat different from the officinal formula; vinegar or sour cider being used in the preparation, thus meeting in some measure the suggestion of Dr. Dewees. The tobacco ointment, itself, is a good application, but the addition of camphor renders it more agreeable to the patient, counteracting, in some measure, the unpleasant odor of the tobacco, and adding probably to its curative powers. It frequently affords relief even after the acute, lancinnating pain and chill, which characterize the onset of the suppurative stage having been developed, and the tumor presents that glazed appearance which precedes the pointing of the abscess. I have a lady under treatment at this time, who has suffered from milk abscess after each of her confinements, (four in number) so that the breast has become entirely useless to her; not being aware of this fact when she came under my care, a few weeks since, my attention was not particularly directed to the breasts, till on one of my visits, I found she had had a chill, followed by fever, and sharp, cutting pains in the left mamma; the gland was much swelled, and just under the nipple was a shining protuberance, indicating the locality for pointing. A muslin cloth, the size of the gland, was spread with the ointment, and the whole surface covered with it immediately—the nipple presenting through an opening in the muslin. In about twenty-four hours, the application being frequently repeated, the pain and induration were partially relieved, and the patient gradually recovered. Within the last two years, I do not recollect to have seen a "gathered breast," though my opportunities for meeting with this form of disease have been four fold, in comparison with any former period—but I have frequently seen in that time, the mammary gland swelled, painful, and threatening suppuration. Constitutional means are not of course omitted in cases where their usual signs are present.—*New Jersey Medical Reporter.—Editorial.*

